# Supplementary material for: Indirect reciprocity with stochastic and dual reputation updates
Source: PLoS Comput Biol. 2023 Jul 20;19(7):e1011271. doi: 10.1371/journal.pcbi.1011271 (PMC10359017; doi:10.1371/journal.pcbi.1011271)
Supplement: Attachment — Submitted filename: reply.pdf [file pcbi.1011271.s001.pdf]

Max Planck Institute for Evolutionary Biology
Plön, Germany
YOHSUKE MURASE
Email: yohsuke.murase@gmail.com

May 15, 2023

Dear Editor,

We are grateful for your consideration of our manuscript entitled “Indirect reciprocity with stochastic and dual reputation updates.” We are pleased that both reviewers evaluated our work quite positively, such as “The findings are significant”, “This manuscript makes several substantial contributions”, “the content of the paper is intriguing and of great interest”. Their inspiring and constructive suggestions are greatly appreciated, and we have tried our best to answer their comments and questions. Here is the list of the major changes we have made in the revised manuscript.

- Inspired by the referee reports, we studied the error sensitivity in more detail and obtained an analytical expression that better supports our conclusion. We added a new section (Section 4.3) and figure (Figure 3) for these results.
- We reorganized the introduction such that the order of the model extensions is consistent with the order of the result sections.
- We discuss the motivation for introducing stochasticity from the viewpoint of possible real-world scenarios.
- We changed the paper’s title to “Indirect reciprocity with stochastic and dual reputation updates,” which reflects the contents of the paper better.

We have also made minor typesetting and typographical corrections to improve the clarity of our manuscript. We hope this revised manuscript is acceptable for publication in Plos Computational Biology.

Yours Sincerely,

Yohsuke Murase
on behalf of the authors

To Reviewer 1

> **Reviewer 1:** This paper explores the topic of indirect reciprocity, introducing two new components for consideration: the updating of player reputations based on other players' actions, and the stochastic updating of reputations. The authors conduct a thorough investigation into the emergence of cooperative equilibria, with a particular focus on invasion barriers. The study reveals the existence of "Secondary Sixteen" norms, which are cooperative ESSes with higher invasion barriers than the classical "Leading Eight" norms; here the benefit of cooperation must be at least twice the cost of cooperation for these norms to be upheld. The authors present a comprehensive list of cooperative ESSes, including the minimum ratio between cooperation benefit and cost for each. The findings are significant, and I recommend the paper for acceptance with minor revisions:

Answer: First of all, we are grateful for your careful reading and constructive suggestions. We are glad to hear that you find our manuscript significant. We have revised our manuscript in accordance with your report.

> **Reviewer 1:** 1. In line 368 (and in Figure 2), it is assumed that $\mu_e = \mu_{a1} = \mu_{a2}$. It would be interesting to see what influence each of the three different error rates has. 2. Similarly, in Subsection 5.3 the benefit-to-cost ratios are examined for some stochastic norms. Here, it would be interesting to see if the trade-off between error robustness and ESS robustness extends to the stochastic norms.

Answer: Inspired by your questions, we studied the error sensitivity more carefully and obtained an approximate analytical expression. The drop of the cooperation level is written as a function of μ_e, μ_{a1}, and μ_{a2}. In short, implementation errors are more harmful than assessment errors because implementation errors cause defections and the subsequent loss of reputation. We numerically verified that these expressions are correct for a large number of randomly generated stochastic norms. We have added a new section (Section 4.3) and figure (Figure 3) for the error sensitivity analysis. Thank you for your inspiring comments.

To Reviewer 2

> **Reviewer 2:** This manuscript makes several substantial contributions to the literature on indirect reciprocity. First, it expands the standard third-order social norms, including the so-called "leading eight", to allow for the possibility that not only is the donor's reputation updated after an interaction but also the recipient's. Second, it identifies a class of deterministic, donor-updating social norms that is a cooperative evolutionary stable state (CESS) for $b/c > 2$, with b the benefit of cooperation and c the cost (in comparison to the leading eight, which are stable for $b/c > 1$); these differ from the "leading eight" insofar as bad recipients defect against good ones but are forgiven anyway. Third, it allows social norms to be stochastic, assigning reputations with certain probabilities (separate from errors). One surprising (and particularly ingenious) finding is that norms that are more robust against errors require higher b/c ratios to sustain stable cooperation. There are several interesting minor results, as well; for example, stochastic version of the S1 "secondary sixteen" norm is almost as "powerful" as the leading eight despite the counterintuitive "bad defects against good but is considered good nonetheless" rule.
> This is all put together in a presentation that is methodical, thorough, and for the most part easy to follow. Sections 2 and 3 function as great outlines for the arguments used in the rest of the paper and provide a (badly needed) intuitive understanding of CESS norms, including the leading eight.

Answer: We are grateful for your careful reading and constructive suggestions. It is our great pleasure that you find our manuscript makes substantial contributions. We are also encouraged to hear that you find our manuscript easy to follow. As shown below, your constructive suggestions helped us improve the manuscript.

> **Reviewer 2:** One, the order of ideas in the abstract and introduction does not match the rest of the paper. Stochastic norms are named as the "first" extension of the standard indirect reciprocity norms; updating the recipient's reputation is named as the "second". But the actual presentation of these ideas is in the reverse order. In fact the paper's title feels a bit odd given that stochastic norms are only really zeroed in on briefly, in section 5.3. I would certainly recommend either re-ordering section 5 to match the order in which these ideas are named or simply swapping the order in which they are listed in the abstract and introduction. The authors could also consider a more general title that better captures the material they spend most of their time on (though I don't have any great ideas offhand; the current title has the advantage of being brief and catchy, and I imagine "Indirect reciprocity in which not only the donor's reputation is updated but also the recipient's" would be hard to compress into five or six words, so this should be taken as a weak suggestion).

Answer: Thank you for pointing this out. When submitting the manuscript, we were thinking along the same lines, but we were unable to find a fully satisfactory title. Your comment, which we fully agree with, made us reconsider. We believe our new title, "Indirect reciprocity with stochastic and dual reputation updates," better reflects the content of this study and is still catchy and concise. We also switched the order in the abstract and intro accordingly. We believe that our revised manuscript is smoother for readers.

> **Reviewer 2:** Two, I would like to see a bit more motivation for stochastic norms. The exploration of stochastic norms is justified solely with reference to indirect reciprocity modeling, but surely there are real-world examples where norms can be modeled as stochastic–for example, averaging over small population differences in social norm, or assessment that depends on the context of an action in a way other than the reputation of the donor and recipient. Probably better motivations can be found. Indirect reciprocity has profound implications for the evolution of human moral systems, so some care to link this part of the paper to said moral systems would be appreciated. By contrast, updating the recipient's reputation is, in the text, well justified with reference to intuition and the literature.

Answer: Thank you for this excellent suggestion as well. We revised the introduction in order to motivate the introduction of stochasticity better. Please see the revised introduction.

> **Reviewer 2:** Third, several of the leading eight are well known because they have catchy names (and because two of them are actually second-order). The authors have the opportunity to coin names for some of the new norms they've derived. Possibly it's hard to come up with punchy names for these; the rule "bad defects with good but is considered good anyway" is counterintuitive and maybe none of these "secondary sixteen" admit a good name. But the authors should at least try.

Answer: This is another thoughtful suggestion. To identify social norms that would be worth having their own names, we looked for norms that are simple to interpret. One of the secondary sixteen is particularly simple. It only assigns a bad reputation when a good donor defects against good recipient. In all other cases, it assigns a good reputation. In particular, bad players automatically receive a good reputation after one round. In the revised manuscript, we thus term this norm “Forgiver”. Thank you for this suggestion.

> **Reviewer 2:** Several extensions are possible (for example, a version of Fig. 2 that considers sensitivity to errors that occur at different rates), but my guess is that they would not provide much more intuition, so I don't think they're really necessary.

Answer: Interestingly, reviewer #1 raised a very similar question, asking us to explore what happens when different error rates can take different values. Fortunately, we obtained an analytical expression for this generalized error sensitivity. The respective formula is now included in the revised manuscript. We also added a figure to show the tradeoff between error robustness and ESS robustness for stochastic norms. See Section 4.3 and Figure 3.

> **Reviewer 2:** There is a typo in equation 4, on the second line (the $h(t)(1-h(t))$ term); the first $\bar{R}_2(B,G)$ wants to be $\bar{R}_1(B,G)$. This error does not appear to have percolated anywhere else in the manuscript (for example equations 7 or 11), but I didn't check all the algebra, so the authors should go through it with a fine-toothed comb just in case.

Answer: Thank you for pointing this out. We have corrected the typo. We also went through the manuscript again and checked our manuscript carefully.
